# The Effect of COVID-19 on the Perioperative Course of Acute Coronary Syndrome in Poland: The Estimation of Perioperative Prognosis and Neural Network Analysis in 243,515 Cases from 2020 to 2021

**DOI:** 10.3390/jcm11185394

**Published:** 2022-09-14

**Authors:** Karol Kaziród-Wolski, Patrycja Zając, Michał Zabojszcz, Agnieszka Kołodziej, Janusz Sielski, Zbigniew Siudak

**Affiliations:** 1 Collegium Medicum, Jan Kochanowski University in Kielce, al. IX Wieków Kielc 19A, 25-369 Kielce, Poland; 2 The Reumatology Department of the Province Hospital in Konskie, ul. Gimnazjalna 41B, 26-200 Konskie, Poland

**Keywords:** COVID-19, acute coronary syndrome, vaccination, myocardial infarction

## Abstract

COVID-19 causes thromboembolic complications that affect the patient’s prognosis. COVID-19 vaccines significantly improve the prognosis for the course of the infection. The aim of this study was to evaluate the impacts of patient characteristics, including COVID-19 vaccinations, on perioperative mortality in acute coronary syndrome in Poland during the pandemic. We analyzed the data of 243,515 patients from the National Registry of Invasive Cardiology Procedures (Ogólnopolski Rejestr Procedur Kardiologii Inwazyjnej [ORPKI]). In this group, 7407 patients (21.74%) had COVID-19. The statistical analysis was based on a neural network that was verified by the random forest method. In 2020, the most significant impact on prognosis came from a diagnosis of unstable angina, a short period (<2 h) from pain occurrence to first medical contact, and a history of stroke. In 2021, the most significant factors were pre-hospital cardiac arrest, female sex, and a short period (<2 h) from first medical contact to coronary angiography. After adjusting for a six-week lag, a diagnosis of unstable angina and psoriasis were found to be relevant in the data from 2020, while in 2021, it was the time from the pain occurrence to the first medical contact (2–12 h) in non-ST segment elevation myocardial infarction and the time from first contact to balloon inflation (2–12 h) in ST-segment elevation myocardial infarction. The number of vaccinations was one of the least significant factors. COVID-19 vaccination does not directly affect perioperative prognosis in patients with acute coronary syndrome.

## 1. Introduction

Numerous studies have demonstrated the link between the SARS-CoV-2 infection, an increased risk of myocarditis and thromboembolic incidents, and the occurrence of acute coronary syndrome or arrhythmia [1,2,3]. The angiotensin-converting enzyme type 2 (ACE-2) receptor and serine proteases (TMPRSS2) have been identified as crucial to the virus’s ability to replicate and fuse with host cells [4]. ACE-2 receptors are downregulated, bringing about the adverse effects of angiotensin, leading to increased metabolic demand, immune activation, microcirculatory dysfunction, and subsequent myocardial damage [2].

Cytokines play an important role in a SARS-CoV-2 infection. Studies on IL-1β, a cytokine with known pro-inflammatory activity, are proving interesting. It appears that blocking this cytokine may reduce myocardial damage and inflammation and may improve oxygenation in COVID-19 patients [5]. Cytotoxins and the systemic inflammatory response affect the endothelium and coagulation system and may generate an increased risk of embolic incidents in COVID-19 patients [6,7]. Pulmonary embolisms may contribute to the development of pulmonary hypertension and secondary right ventricular failure. In one study, subclinical right ventricular dysfunction was found in 42% of post-COVID-19 patients [8].

During the COVID-19 pandemic, the course of ischemic heart disease was influenced by numerous classical factors. Their distribution in the population was similar to the time before the pandemic [9]. Vaccinations have increased the incidences of myocarditis and pericarditis, especially in the younger population [10]. As the symptoms reported by patients (i.e., chest pain) are similar, this could result in some difficulties in the final selection of patients referred for invasive diagnostics [11].

With the increasing number of cases, a race against time took place to develop an effective vaccine against the viral infection. The COVID-19 vaccination program began in December 2020. Data on vaccinated individuals are continuously being updated. As of May 2022, more than five billion people worldwide had received at least one dose of a vaccine [12]. In Poland, in 2022, the daily number of vaccinations ranged from 305,900 in January to 7800 in July 2022 [13].

To determine whether COVID-19 vaccinations affected the perioperative course in patients with acute coronary syndrome (ACS) in Poland, we analyzed the available data via a neural network model. Technological advances have contributed to the development of artificial intelligence with machine learning and deep learning mechanisms based on multilayer neural networks. The first mathematical model of a neuron, in the form of an arithmetical/logical system, was proposed by McCulloch and Pitts in 1943. It was an innovative work [14], the concept of which survives to this day and forms the basic building blocks of the perceptron neural network. Neural networks are modeled on the structure and operation of neurons in the human brain. Artificial neural networks typically consist of three layers: an input layer, a hidden layer, and an output layer. The most common type of neural network unit computes a weighted sum of input data and transforms the result nonlinearly [15,16]. As with human beings, neural networks learn on the basis of examples—more precisely, on data sets—which are created using appropriate learning algorithms. There are two basic types of learning: supervised and unsupervised. The widespread use of neural networks has become possible with the development of computer programs. Nowadays, they are used in many fields outside of medicine, including economics, automation, and energy technology.

The purpose of this study was to evaluate whether the increasing vaccination rates during the first two years of the pandemic (2020 and 2021) affected the underlying factors that determine the course of ACS and perioperative death.

## 2. Material and Methods

The aim of the presented study was to determine the impact of clinical factors extracted from the synthesis of large databases on short-term prognosis (perioperative death) using a neural network.

For this study, we used data from the National Registry of Invasive Cardiology Procedures (*Ogólnopolski Rejestr Procedur Kardiologii Inwazyjnej* [ORPKI]). The centers of invasive cardiology in Poland that are associated with the registry collect the data electronically. Currently, this includes 161 catheterization labs in Poland. Before the pandemic, patients with ACS were managed according to the guidelines of the Polish Cardiac Society. 

Data on the clinical course of ACS and concomitant conditions were obtained from the patient interviews and records from the emergency room (with admitting physicians and hemodynamics laboratory physicians). The ORPKI registry included all patients admitted with ACS; the obtained variables were automatically entered. The outbreak of the epidemic, and then the pandemic, brought about different approaches regarding patients who required invasive treatment but were also infected with COVID-19. This type of management is consistent with the principles of invasive treatment as well as self-protection and the protection of uninfected patients in the same hospital.

Patients who underwent invasive treatment for ACS in 2020 and 2021 were included in the study group. The total number of evaluated patients, i.e., patients who were qualified for ACS treatment and registered in ORPKI, was 243,515. This group consisted of 190,595 patients without COVID-19 (78.26%) and 7407 patients who had been diagnosed with COVID-19 (21.74%). We analyzed the latter group. A positive result was determined by an antigen test performed in the ambulance or at the destination hospital. Due to limitations of time, polymerase chain reaction (PCR) test results were not relied upon. Patients with suspected COVID-19 infections (as recommended for triage by the National Institute of Public Health and the Ministry of Health) were treated as potentially COVID-19 (+). A diagnosis of COVID-19 was always available before any intervention (angiography or percutaneous coronary intervention) and recorded in the ORPKI online database. Samples for molecular RT-PCR were always obtained before the procedure.

COVID-19 infections were confirmed by RT-PCR tests in 2020 and by rapid antigen tests and/or RT-PCR tests in 2021. The oxygen saturation of the patient was measured by a non-invasive method before qualification. STEMI with a short duration of ischemia was qualified for immediate intervention, while NSTEMI and UA underwent comprehensive evaluation. The optimal pathway and timing of the intervention were chosen. If the patient had features of respiratory failure, then s/he was referred for ventilatory support treatment. Patients with ACS (from all over the country) were eligible for the study. Laboratory tests were performed after the invasive procedure; the authors did not have access to the results. This is beyond the scope of the current study. 

The study analyzed the impact of the daily number of vaccinations in the voivodeship where the ACS occurred based on official data [13]. Descriptive characteristics included the average daily number of vaccinations for both COVID-19 + and −.

We performed a pooled analysis of comorbidities, predisposing factors, and medications in patients with ACS and COVID-19. Two groups of patients were compared: patients with ACS but no confirmed infection, i.e., COVID-19 (−), and patients with ACS and confirmed infection, i.e., COVID-19 (+). Patients who qualified for invasive treatment signed informed consent forms in accordance with the recommendations of the 1964 Helsinki Declaration. Since we used anonymous data from the ORPKI database, the study did not require the approval of the Bioethics Committee. 

In-hospital-perioperative death was the primary endpoint of our study. Since the ORPKI database aims to collect data on patients until invasive treatments are implemented, it was not possible to continue patient follow-up after leaving the Cath Lab. Thus, it was not possible to determine the number of deaths in intermediate and long-term observations. Perioperative death is, according to the authors, a good reflection of the impacts of many factors during prehospital management on patient prognosis. UA is defined as the occurrence of sudden angina symptoms or a significant exacerbation present without an increase in infarction markers [17].

## 3. Statistical Methods

The study sample was randomly divided into two groups: training (70%) and validation (30%). Two models were used to compare the results of predicting death during the procedure. The first model was a regression feedforward fully connected multilayer perceptron neural network with three hidden layers. All 35 variables were added to the input layer. During the learning process, each patient was randomly presented as a new learning case. The algorithm repeatedly attempted to match the variable weights to obtain the best prediction of the outcome. One numerical variable to be predicted—the number of deaths during the procedure aggregated by day and voivodeship (unit of administrative division of the highest level in Poland)—was in the output layer. Three hidden layers were constructed between the input and output layers, which allowed more complicated patterns to be identified between the input and output variables. We tuned the neural network’s hyperparameters in an empirical, experimental way. The rectifier linear unit (ReLU) activation function was used. The mean squared error loss was used for neural network optimization. The neural network was trained with backpropagation using an adaptive stochastic gradient descent algorithm. The model was evaluated with a loss curve, prediction vs. target plot, and mean squared error. After the neural network was trained and evaluated, the permutation importance analysis was performed to determine which variable had the greatest impact on the neural network model. The results are presented as a graph. The model was trained independently four times. The training was conducted separately for the 2020 data (without the impact of vaccines) and the 2021 data (with the impact of vaccines). Moreover, training was conducted separately for datasets with immediate vaccination effects (to check for the immediate vaccination effects on death during the procedure) and six-week delayed vaccination effects (to check—after six weeks—whether patient vaccinations had an impact on deaths during the procedures).

The second model was a regression random forest model. All 35 variables were added as inputs. During the learning process, each patient was randomly presented as a new learning case. The algorithm repeatedly created a decision tree for that sample for the assigned number of trees. Then, an average across all decision trees was calculated and taken as the final prediction. Thus, the mean prediction of the one numerical variable—the number of deaths during the procedure aggregated by day and voivodeship—was the model’s output. We tuned the random forest hyperparameters in an empirical, experimental way. The squared error criterion was used. One hundred decision trees were constructed for each random sample, which allowed more complicated patterns to be identified between the input variables and the output variable. The model was evaluated with a loss curve, prediction vs. target plot, and mean squared error. After the random forest was trained and evaluated, the Gini importance analysis was performed to determine which variable had the greatest impact on the random forest model. The results are presented as a graph. The model was trained independently four times. The training was conducted separately for the 2020 data (without the impact of vaccines) and the 2021 data (with the impact of vaccines). Moreover, training was conducted separately for datasets with immediate vaccination effects (to check for immediate vaccination effects on deaths during the procedure) and with six-week delayed vaccination effects (to check—after six weeks—whether patient vaccinations had an impact on deaths during the procedure).

Probabilistic modeling, together with artificial neural networks, is called the “Bayesian neural network”. In both artificial neural and Bayesian networks, the random initial values of parameters were sampled from probability distributions, e.g., normal. However, during training, in Bayesian neural networks, the probability distribution of each network parameter is modeled; the single values themselves are not adjusted, as in a classical neural network. Treating parameters as probabilistic would prevent changes to single parameter values during repeated training of the model, although the random root in deep learning is still in many places; it is likely that random fluctuations in single values would turn into fluctuations in network probability distributions. There is also an approach where the input and output variables are treated as probability distributions, they are probabilistic neural networks.

It is not certain whether such a procedure could affect the lack of random fluctuations in the significance of variables for the model; perhaps random fluctuations in the significance of individual variables (based on the values of these variables) would turn into random fluctuations (in the significance of individual probability distributions of variables). However, articles are available in the literature that present Bayesian statistics in such calculations [18].

## 4. Results

**Figure 1:**
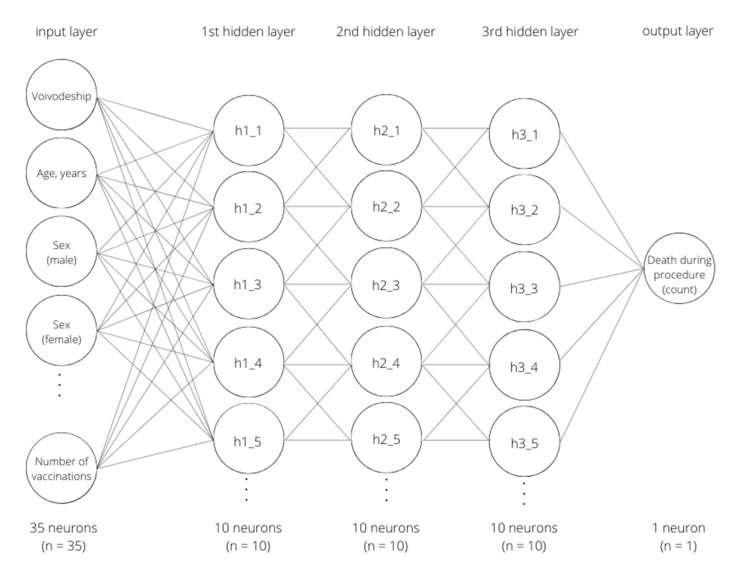
Model of the neural network used in the study. This model is a deep artificial neural network (DNN) with architecture, consisting of 35 input neurons (for one variable each), 3 hidden layers with 10 neurons each (for feature extraction from data), and 1 output neuron (for the prediction of the count of patient deaths during the procedures).

The descriptive characteristics revealed multiple differences between the COVID-19 (−) and COVID-19 (+) groups (Table 1 and Table 2). The authors used a fully-connected multilayer perceptron neural network with three hidden layers (Figure 1). 

**Figure 2:**
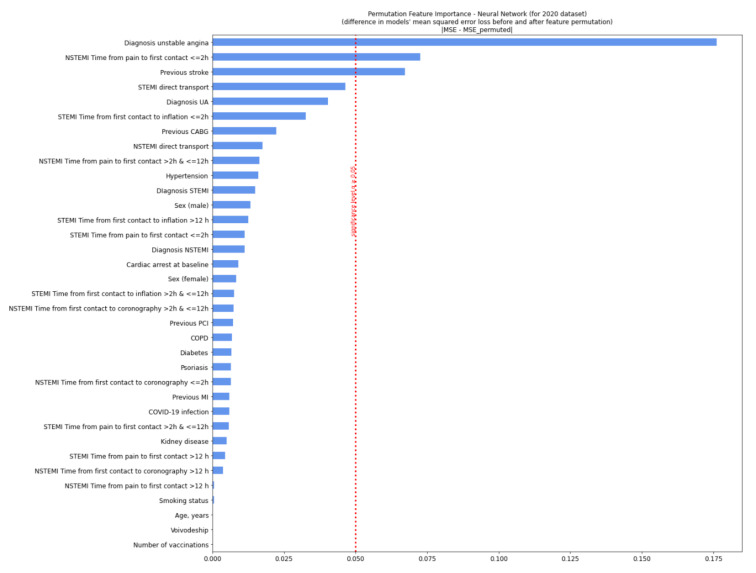
Permutation feature importance analysis for patients in 2020. The diagram shows a decrease in the model’s efficiency when values in single individual features are randomly shuffled column-wise and the rest of the features are left unchanged. We measured neural network efficiency by calculating the mean squared error (MSE) between the target value (true number of deaths during the procedure) and the predicted value (model’s output). The calculation was conducted for each row from data separately and then averaged. In permutation feature importance, the difference between the MSE error from the neural network, predicting the value from the non-preprocessed dataset, and the MSE error from the neural network—predicting the value from the permuted dataset was measured and showed a decrease. Significance *p* ≥ 0.05. CABG—coronary artery bypass graft; COPD—chronic obstructive pulmonary disease; MI—myocardial infarction; NSTEMI—non-ST-elevation myocardial infarction; PCI—percutaneous coronary intervention; Q—quartile; STEMI—ST-elevation myocardial infarction; UA—unstable angina (defined as the occurrence of sudden angina symptoms or a significant exacerbation present without an increase in infarction markers).

**Figure 3:**
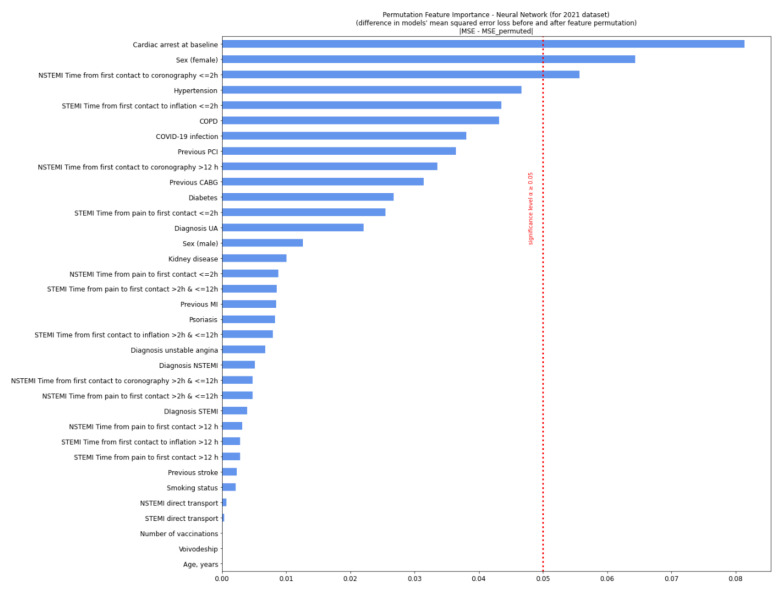
Permutation feature importance analysis for patients in 2020. The diagram shows a decrease in the model’s efficiency when values in single individual features are randomly shuffled column-wise and the rest of the features are left unchanged. We measured neural network efficiency by calculating the mean squared error (MSE) between the target value (true number of deaths during the procedure) and the predicted value (model’s output). The calculation was conducted for each row from data separately and then averaged. In permutation feature importance, the difference between the MSE error from the neural network, predicting the value from the non-preprocessed dataset, and the MSE error from the neural network—predicting the value from the permuted dataset was measured and showed a decrease on the plot. Variables, when permuted, were affected most in the model’s efficiency decrease and exceeded the significance threshold. Significance *p* ≥ 0.05. CABG—coronary artery bypass graft; COPD—chronic obstructive pulmonary disease; MI—myocardial infarction; NSTEMI—non-ST-elevation myocardial infarction; PCI—percutaneous coronary intervention; Q—quartile; STEMI—ST-elevation myocardial infarction; UA—unstable angina.

For the 2020 variables, this model showed that the most relevant variables for prognosis were a diagnosis of unstable angina, a short period from pain occurrence to first medical contact (<2 h), and a history of stroke. For 2021, the relevant variables were pre-hospital cardiac arrest, female sex, and a short period from first medical contact to coronary angiography (<2 h) (Figure 2 and Figure 3). The model was of good quality (Supplementary Figures S1–S4). After adjusting for a six-week lag, the prognostically relevant factors were the diagnosis of unstable angina and psoriasis (2020).

**Figure 4:**
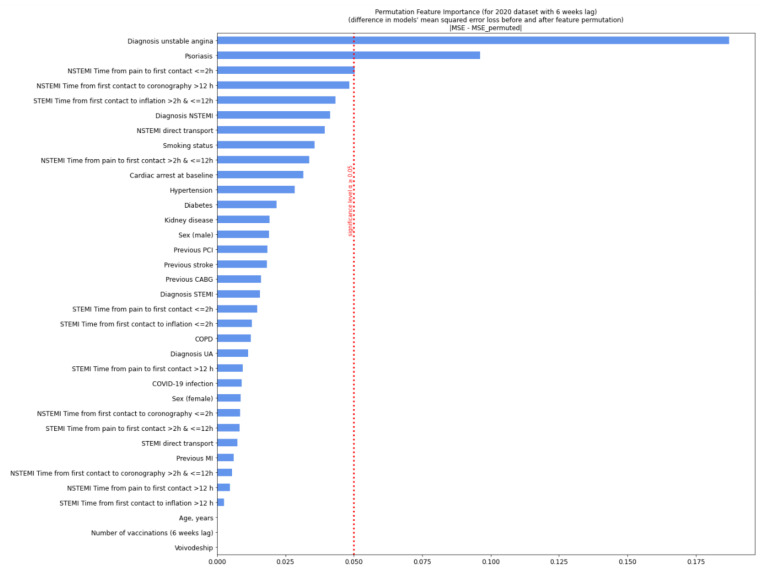
Permutation feature importance analysis for patients in 2020 with a six-week lag. Diagram shows the decrease in the model’s efficiency when values in single individual features are randomly shuffled column-wise and the rest of the features are left unchanged. We measured the neural network efficiency by calculating the mean squared error (MSE) between the target value (true number of deaths during the procedure) and the predicted value (model’s output). The calculation was conducted for each row from data separately and then averaged. In the permutation feature importance, the difference between the MSE error from the neural network, the predicting value from the non-preprocessed dataset, and the MSE error from the neural network, the predicting value from the permuted dataset was measured and showed a decrease on the plot. Variables, when permuted, were affected most in the model’s efficiency decrease and exceeded the significance threshold. Significance *p* ≥ 0.05. CABG—coronary artery bypass graft; COPD—chronic obstructive pulmonary disease; MI—myocardial infarction; NSTEMI—non-ST-elevation myocardial infarction; PCI—percutaneous coronary intervention; Q—quartile; STEMI—ST-elevation myocardial infarction; UA—unstable angina.

**Figure 5:**
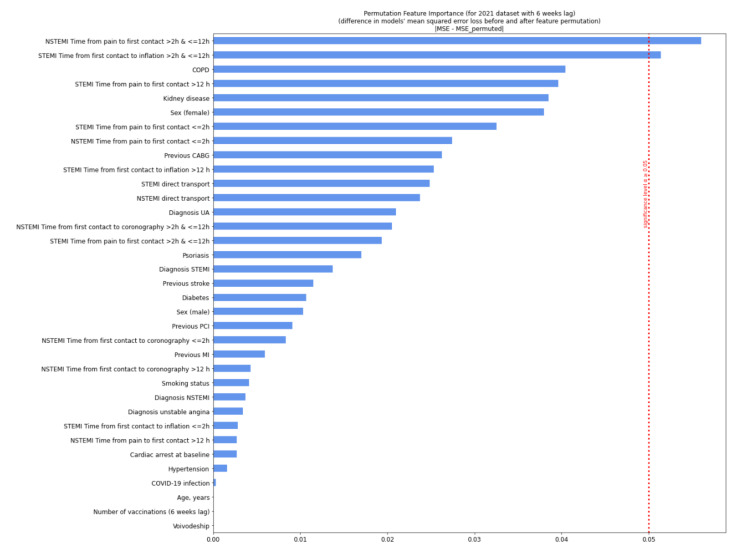
Permutation feature importance analysis for patients in 2021 with a six-week lag. Diagram shows a decrease in the model’s efficiency when values in single individual features are randomly shuffled column-wise and the rest of the features are left unchanged. We measured the neural network efficiency by calculating the mean squared error (MSE) between the target value (true number of deaths during the procedure) and the predicted value (model’s output). The calculation was conducted for each row from data separately and then averaged. In permutation feature importance, the difference between the MSE error from the neural network, the predicting value from the non-preprocessed dataset, and the MSE error from the neural network, the predicting value from the permuted dataset was measured and showed a decrease on the plot. Variables, when permuted, were affected most in the model’s efficiency decrease and exceeded the significance threshold. Significance *p* ≥ 0.05. CABG—coronary artery bypass graft; COPD—chronic obstructive pulmonary disease; MI—myocardial infarction; NSTEMI—non-ST-elevation myocardial infarction; PCI—percutaneous coronary intervention; Q—quartile; STEMI—ST-elevation myocardial infarction; UA—unstable angina.

In 2021, the most relevant factors were the time from pain occurrence to first medical contact (2–12 h) in non-ST segment elevation myocardial infarction (NSTEMI) and the time from first contact to balloon inflation (2–12 h) in ST-elevation myocardial infarction (STEMI) (Figure 4 and Figure 5). 

The quality of the model was good (Supplementary Figures S5–S8). Using the second random forest model, no variables were found relevant in 2020 and 2021 (Figure 6 and Figure 7), even considering a six-week delay (Figure 8 and Figure 9). 

The quality of the model was good (Supplementary Figures S9–S12). In each model, vaccination was the least relevant factor affecting the occurrence of periprocedural death.

## 5. Discussion

The COVID-19 pandemic had a profound impact on healthcare around the world. The different approaches to COVID-19 (+) patients required a number of changes that could be observed as successive waves of infections passed. Healthcare organizations had to be versatile in order to safeguard patients with acute conditions, including those with ACS.

Kaziród-Wolski et al. compared COVID-19 (+) and COVID-19 (−) patients treated for ACS in 2020 and found that COVID-19 (+) patients were more frequently transported to the interventional cardiology laboratory within 12 h of symptom onset than COVID-19 (−) patients. This indicates an adequate organization of emergency and invasive cardiology services and efficient healthcare organization [9]. Similar results were published by Matsushita et al., who found minimal delay before treating patients with STEMI and COVID-19 infections [19].

Patients with COVID-19 and ACS have worse prognoses [9], which may be caused by different pathophysiology states during the course of the disease. SARS-CoV-2 has the ability to damage cells directly or indirectly by inducing excessive activation of the coagulation system, an abnormal systemic inflammatory response, or endothelial dysfunction [6,7]. Further research in this area may contribute to more effective treatment of COVID-19 patients.

Infections caused by SARS-CoV-2 are associated with an increased risk of cardiovascular complications. Katsoularis et al. estimated the risk of ACS to be higher in COVID-19 (+) patients than in the control group in a study involving a large group of patients. For ACS, excluding the day of exposure, the incidence rate ratio (IRR) was 2.53 (1.29–4.94) and the odds ratio (OR) in the control group was 3.41(1.58–4.94) at week 2 of COVID-19 infection. For the analysis, including day 0, the IRR was 2.56 (1.31–5.01) and the OR was 6.61(3.56–12.20) [1].

The analysis of our study population revealed that periprocedural mortality was associated with sudden pre-hospital cardiac arrest, a diagnosis of unstable angina, and NSTEMI with the onset of pain occurring 2 to 12 h after initial medical contact. Previous studies reported an association between vaccination and the course of ACS. Showkathali et al. conducted a study on 89 patients with a diagnosis of ACS and the presence of coronary thrombus on coronary angiography. In this group, 37 patients (42%) had been vaccinated against COVID-19 (with COVISHIELD and Covaxin vaccines) one to four weeks earlier [20]. Similar observations regarding the presence of thrombi in the coronary arteries and the likely association with COVID-19 vaccination were reported by Tajstra et al. In a case report of an 86-year-old patient with ACS, three thrombi were found in the coronary arteries, likely associated with COVID-19 vaccination. This patient was administered the Pfizer-BioNTech vaccine [21]. In our study of a large sample (n= 243,515), there was no significant association between periprocedural death during the course of ACS and vaccination against COVID-19 in the shorter (four weeks) or longer (six weeks) periods. 

In the fight against the COVID-19 pandemic, the relevance of vaccines has been emphasized. While they provide varying levels of protection, they have shown great efficacy in preventing severe disease course, hospitalization, or death caused by infection. So far, several vaccine types have been developed. They induce adequate immune responses through different mechanisms. According to studies conducted on the immune response after vaccination against COVID-19, the first measurable level of antibodies in individuals without prior infection occurs 14 to 28 days after the first vaccine dose [20]. The second dose results in a significant increase in antibody titers, with a peak occurring several days after vaccination [22]. The post-vaccine immune response varies according to the type of vaccine, the number of doses, the person’s age, and whether they were infected before the first dose of vaccination. In convalescent patients, the increase in antibody levels is greater after the first dose [22,23].

A recurring topic that usually accompanies the discussion of vaccinations is adverse reactions. In the case of the most common vaccines, headache, fatigue, pain at the injection site, nausea, and muscle pain are among the most frequently reported adverse reactions. In terms of the effects of vaccines on the cardiovascular system, there has been some discussion about the complication of myocarditis caused by mRNA vaccines. A large meta-analysis estimated the incidence of such a complication at 11 per 10,000 vaccinated individuals [24]. In another study, the overall incidence of myopericarditis after vaccination against COVID-19 in 22 studies was 33.3 cases (95% CI, 15.3–72.6) per million doses of vaccine [25]. Thus, the incidences of such complications are rare. 

In the treatment of myocarditis during COVID-19, steroid therapy may yield favorable results; the use of other agents, such as IL-6 inhibitors, intravenous immune globulin, and colchicine, seems questionable [26].

For several years, the authors conducted studies on large groups of patients and cardiac registries. These studies used perioperative death as an endpoint [27,28]. The COVID-19 pandemic, which occurred between 2020 and 2021, caused significant changes in the healthcare system and patient management (including ACS patients being eligible for invasive treatment). The pandemic has become a new element in the evaluation of ACS patients. Morbidity and the vaccination program may also be elements of this evaluation.

In Poland, during the first year of the pandemic, the ACS number decreased significantly [29]. We could speculate that patients did not want to report to hospitals during the peak of the pandemic. On the other hand, the organizational problems with access to specialized care could have been an obstacle. During the second year of the analysis, a relative normalization of the situation was observed. Differences due to the pandemic were unavoidable. However, in our opinion, they did not affect the results of the neural network analysis because each year one individual factor was analyzed separately. The results obtained allow us to faithfully reproduce the course of the ACS in the first two years of the pandemic.

After adjusting for the six-week post-vaccination period, psoriasis proved to be a prognostically significant factor in our study. The impact of psoriasis on the prognosis of patients with ACS has already been described. Psoriasis is a systemic inflammatory disease [30] and has implications beyond the skin. Studies have demonstrated the impact of psoriasis on the cardiovascular system, with the risk of death from cardiovascular causes estimated to be 57% higher in patients with severe psoriasis [31]. Other authors have indicated an increased risk of myocardial infarction (HR: 1.21) in patients with severe psoriasis [32]. An attempt to explain the correlation of psoriasis with increased atherosclerosis and increased risk of coronary artery disease is the fact that both conditions show similarities in the immunoinflammatory mechanisms related to the activation of T-helper Th1 and Th17 cells and decreased regulatory T cell function [32]. To date, data on psoriasis and ACS during the COVID-19 pandemic and their impacts on these conditions are limited. However, it appears that the prevailing inflammation that underlies the course of these three diseases may interact with each other and produce an exacerbated effect. It has been proven that patients with COVID-19 experience exacerbated psoriasis symptoms [33]. Interesting data were provided by a randomized, double-blind, placebo-controlled study, where secukinumab was used in patients with psoriasis. It was shown that such treatment may have a beneficial effect on cardiovascular risk by improving endothelial functions [34]. The endothelium and the degree of its damage is one of the key elements of pathophysiology in COVID-19. Our study indicates the need for further research on the impact of psoriasis on CVD risk in patients with COVID-19.

Differences in the clinical course of different forms of ischemic heart disease during the COVID-19 pandemic are well known and described. Hakim R. et al. presented a 25% reduction in invasive treatment in STEMI from January to April 2020 (at the beginning of the pandemic) compared to the previous year in a large study involving the French registry [35]. Similarly, other researchers in their observations reported a significant reduction in invasive treatment in STEMI during the COVID-19 pandemic. In Austria—25.5%, Italy—26.5%, Spain—40%, the United States—48%, and China—26% [36,37,38,39,40]. 

There were also significant differences in the course of UA during the pandemic. We found the differences in diagnoses affecting patient prognoses between the years. In 2020, these were unstable angina, a short period from pain onset to the first medical contact (<2 h), and history of stroke; in 2021, they were pre-hospital cardiac arrest, female gender, and the short period from first medical contact to coronary angiography (<2 h). One particularly interesting finding seems to be the impact of the pandemic on the course of angina and its effect on patient prognosis. Possible explanations for this observation may be related to the healthcare system as well as the patient himself. Desai et al. studied the course of cardiac catheterization for suspected ACS or OHCA between January 1, 2020, and June 2, 2020, which was divided into three subgroups: pre-isolation, strict isolation, and loosened isolation periods. During the period indicated as “pre-isolation” to “strict isolation period,” there was a significant reduction in the mean weekly number of catheterizations for NSTEMI/UA (8.29 vs. 12.5, *p* = 0.019), while the mean weekly catheterization events for NSTEMI/UA increased by 27% between the “strict isolation” and “loosened isolation period,” which the authors explain as a “rebound effect” [41]. Therefore, we hypothesize that a similar phenomenon and accumulation of UA cases may have occurred in the case of our observations. An additional factor contributing to such an effect may include emotional stress, especially during the pandemic. It has been shown that stress, through the generation of hyperglycemia, in patients without previously diagnosed diabetes, was associated with a poorer prognosis in patients admitted to the hospital for ACS [42] and, therefore, may have influenced the different courses of UA. Our 2021 data differed from the previous year. Perhaps this was influenced by a period of relative stability, the implementation of appropriate patient management procedures, mass vaccinations, and the natural evolution of SARS-CoV-2. Thus, the different incidences of UA and STEMI in our study can be explained by the atypical incidences of UA and STEMI during the COVID-19 pandemic period compared to the pre-pandemic period. 

Even though the research is ongoing, preliminary data show significant benefits from vaccination. However, the problem may be the emergence of new variants of SARS-CoV-2 and the limited efficacy of the immune response generated by vaccines.

The current study compared patients with and without COVID-19 who were diagnosed with ACS. Neural analysis revealed no significant effect of COVID-19 vaccination on perioperative mortality in ACS patients. 

Neural networks are stochastic algorithms (until training is complete). Neural network training involves many random elements, e.g., the initial initialization of parameters [43], the random order in which individual rows from the training databases are presented to the network, the random division between the training and validation datasets, and the detailed random aspects of the optimization algorithms. All of these factors have led to the problem of reproducibility and reliability of analyses using neural networks.

The parameters (weights) of the neural network, which were initialized randomly, were overwritten accordingly during the training of the network, to minimize the network’s error and bring the values of the network’s predictions closer to the known true values. Since the weights were permutable in terms of hidden layers, there were many different solutions (multiple minima) that led to the same low network error. This means that neural networks are non-convex in terms of the objective [44]. Moreover, different combinations of the model parameters themselves, such as the learning rate, the type of optimizer, or the number of hidden layers, can lead to the same, equally good result. This means the under-specification of neural networks [45]. In addition, quantization errors and the types of hardware used in the calculations contribute to randomness [46].

Regarding preprocessing the data before using them to train the neural network, and then the model construction itself—training and obtaining results in the deep learning domain is a process that combines the application of many different computational tools that have different accuracies [47]. Differences in the accuracy of the tools also affect the randomness and, thus, the non-reproducibility of subsequent experiments on neural network models.

## 6. Conclusions

According to our neural network study, the increasing number of patients with COVID-19 did not affect periprocedural death in ACS. Classic clinical factors had the greatest impact on the occurrences of perioperative death in ACS during the COVID-19 pandemic. The estimations of periprocedural death through neural networks and statistical models were useful for analyzing large amounts of data and population variables.

## 7. Study Limitations

The results show factors that are not consistent with large clinical experience, e.g., UA is a greater risk factor for death than STEMI and psoriasis is a leading risk factor for death. Moreover, the inconsistency between 2020 and 2021 raises significant concerns about the reliability and reproducibility of the neural network approach. 

No information was obtained about the type of vaccine administered or the number of doses given to individual patients. Another limitation of the presented study was the lack of determination of the severity of psoriasis.

## Figures and Tables

**Figure 6:**
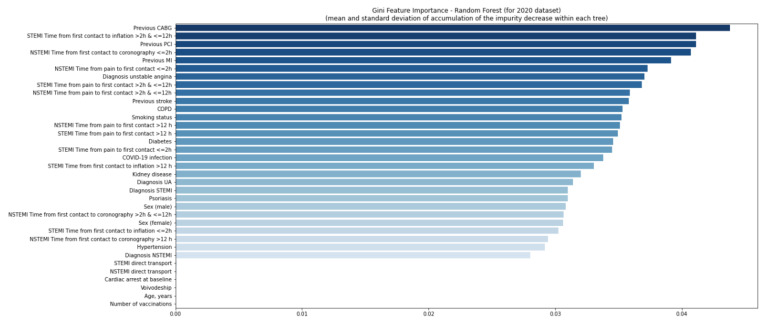
Gini feature importance analysis for patients in 2020. The diagram shows the mean decrease of the model’s impurity. In the analysis, each feature’s importance is calculated as the sum over the number of splits across all trees, which includes the features proportional to the number of samples in the split. In the random forest model, the variable is considered important if the tree split on this variable is affected by large impurity (labels homogeneity in the node) and decreases. CABG—coronary artery bypass graft; COPD—chronic obstructive pulmonary disease; MI—myocardial infarction; NSTEMI—non-ST-elevation myocardial infarction; PCI—percutaneous coronary intervention; Q—quartile; STEMI—ST-elevation myocardial infarction; UA—unstable angina.

**Figure 7:**
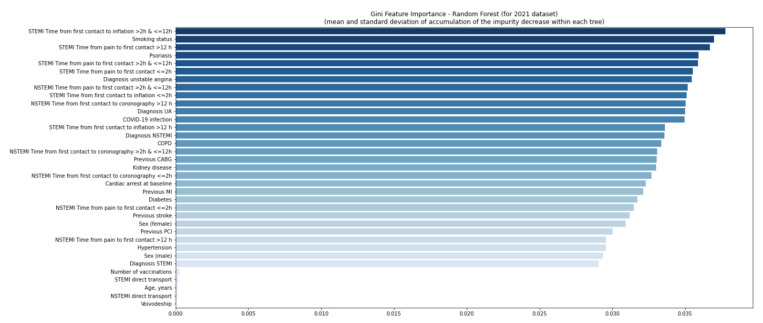
Gini feature importance analysis for patients in 2021. Diagram shows the mean decrease of the model’s impurity. In the analysis, each feature’s importance is calculated as the sum over the number of splits across all trees, which includes the feature proportional to the number of samples in the split. In the random forest model, the variable is considered important if the tree split on this variable is affected by large impurity (labels homogeneity in the node) and decreases. CABG—coronary artery bypass graft; COPD—chronic obstructive pulmonary disease; MI—myocardial infarction; NSTEMI—non-ST-elevation myocardial infarction; PCI—percutaneous coronary intervention; Q—quartile; STEMI—ST-elevation myocardial infarction; UA—unstable angina.

**Figure 8:**
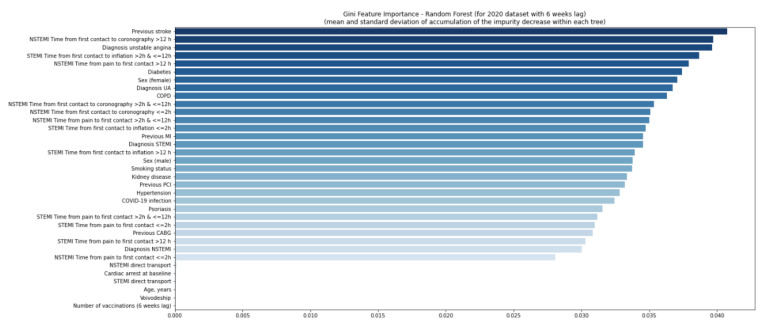
Gini feature importance analysis for patients in 2020 with a six-week lag. The diagram shows the mean decrease of the model’s impurity. In the analysis, each feature’s importance is calculated as the sum over the number of splits across all trees, which includes the feature proportional to the number of samples in the split. In the random forest model, the variable is considered important if the tree split on this variable is affected by large impurity (labels homogeneity in the node) and decrease. CABG—coronary artery bypass graft; COPD—chronic obstructive pulmonary disease; MI—myocardial infarction; NSTEMI—non-ST-elevation myocardial infarction; PCI—percutaneous coronary intervention; Q—quartile; STEMI—ST-elevation myocardial infarction; UA—unstable angina.

**Figure 9:**
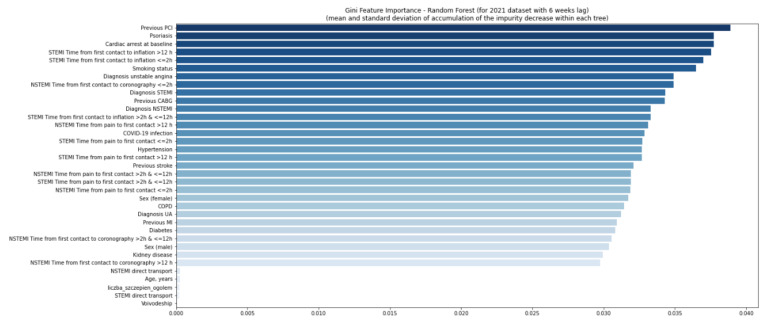
Gini feature importance analysis for patients in 2020 with a six-week lag. The diagram shows the mean decrease of the model’s impurity. In the analysis, each feature’s importance is calculated as the sum over the number of splits across all trees, which includes the feature proportional to the number of samples in the split. In the random forest model, the variable is considered important if the tree split on this variable is affected by large impurity (labels homogeneity in the node) and decreases. CABG—coronary artery bypass graft; COPD—chronic obstructive pulmonary disease; MI—myocardial infarction; NSTEMI—non-ST-elevation myocardial infarction; PCI—percutaneous coronary intervention; Q—quartile; STEMI—ST-elevation myocardial infarction; UA—unstable angina.

**Table 1:** Characteristics of patients analyzed in 2020.

	Missing	Overall	COVID-19 (−)	COVID-19 (+)	*p*-Value
*n*		78,689	50,710	3104	
Age, years, median [Q1, Q3]	52	67.0 [60.0, 74.0]	67.0 [60.0, 74.0]	66.0 [60.0, 74.0]	0.001
Sex (male), *n* (%)	454	50,704 (64.8)	32,850 (64.9)	2150 (69.7)	<0.001
Diabetes, *n* (%)	0	17,204 (21.9)	11,356 (22.4)	662 (21.3)	0.173
Previous stroke, *n* (%)	0	2400 (3.0)	1516 (3.0)	110 (3.5)	0.090
Previous MI, *n* (%)	0	19,918 (25.3)	13,410 (26.4)	518 (16.7)	<0.001
Previous PCI, *n* (%)	0	23,332 (29.7)	15,657 (30.9)	507 (16.3)	<0.001
Previous CABG, *n* (%)	0	3972 (5.0)	2537 (5.0)	107 (3.4)	<0.001
Smoking status, *n* (%)	0	16,880 (21.5)	11,018 (21.7)	762 (24.5)	<0.001
Hypertension, *n* (%)	0	52,579 (66.8)	34,288 (67.6)	1847 (59.5)	<0.001
Kidney disease, *n* (%)	0	4301 (5.5)	2914 (5.7)	182 (5.9)	0.817
COPD, *n* (%)	0	2614 (3.3)	1729 (3.4)	125 (4.0)	0.075
Diagnosis STEMI, *n* (%)	0	17,989 (22.9)	11,468 (22.6)	1469 (47.3)	<0.001
Diagnosis NSTEMI, *n* (%)	0	22,372 (28.4)	14,505 (28.6)	966 (31.1)	0.003
Diagnosis UA, *n* (%) *	0	34,195 (43.5)	21,958 (43.3)	636 (20.5)	<0.001
Diagnosis unstable angina, *n* (%) *	0	4133 (5.3)	2779 (5.5)	33 (1.1)	<0.001
STEMI direct transport, *n* (%)	8457	4609 (6.6)	2895 (6.4)	548 (18.3)	<0.001
NSTEMI direct transport, *n* (%)	8457	1190 (1.7)	727 (1.6)	89 (3.0)	<0.001
Cardiac arrest at baseline, *n* (%)	8457	945 (1.3)	489 (1.1)	237 (7.9)	<0.001
Death during the procedure, *n* (%)	0	276 (0.4)	178 (0.4)	27 (0.9)	<0.001
STEMI time from pain to first contact ≤ 2 h, *n* (%)	0	7114 (9.0)	4491 (8.9)	706 (22.7)	<0.001
STEMI time from pain to first contact > 2 h and ≤12 h, *n* (%)	0	13,844 (17.6)	8828 (17.4)	1263 (40.7)	<0.001
STEMI time from pain to first contact > 12 h, *n* (%)	0	1958 (2.5)	1240 (2.4)	177 (5.7)	<0.001
STEMI time from first contact to inflation ≤ 2 h, *n* (%)	0	10,047 (12.8)	6476 (12.8)	909 (29.3)	<0.001
STEMI time from first contact to inflation > 2 h and ≤12 h, *n* (%)	0	13,583 (17.3)	8674 (17.1)	1269 (40.9)	<0.001
STEMI time from first contact to inflation > 12 h, *n* (%)	0	418 (0.5)	269 (0.5)	34 (1.1)	<0.001
NSTEMI time from pain to first contact ≤ 2 h, *n* (%)	0	4824 (6.1)	3130 (6.2)	224 (7.2)	0.022
NSTEMI time from pain to first contact > 2 h and ≤12 h, *n* (%)	0	15,448 (19.6)	10,029 (19.8)	702 (22.6)	<0.001
NSTEMI time from pain to first contact > 12 h, *n* (%)	0	15,108 (19.2)	9826 (19.4)	676 (21.8)	0.001
NSTEMI time from first contact to coronarography ≤ 2 h, *n* (%)	0	3214 (4.1)	2007 (4.0)	224 (7.2)	<0.001
NSTEMI time from first contact to coronarography > 2 h and ≤12 h, *n* (%)	0	15,562 (19.8)	10,062 (19.8)	780 (25.1)	<0.001
NSTEMI time from first contact to coronarography > 12 h, *n* (%)	0	5460 (6.9)	3677 (7.3)	199 (6.4)	0.085
Number of vaccinations, mean (SD)	0	0.0 (0.0)	0.0 (0.0)	0.0 (0.0)	0

CABG—coronary artery bypass graft; COPD—chronic obstructive pulmonary disease; MI—myocardial infarction; NSTEMI—non-ST-elevation myocardial infarction; PCI—percutaneous coronary intervention; Q—quartile; STEMI—ST-elevation myocardial infarction; UA—unstable angina; * variables counted separately by the computer algorithm (but they should be interpreted together).

**Table 2:** Characteristics of patients analyzed in 2021.

	Missing	Overall	COVID-19 (−)	COVID-19 (+)	*p*-Value
*n*		164,826	139,885	4303	
Age, years, median [Q1,Q3]	90	68.0 [61.0, 74.0]	68.0 [61.0, 74.0]	67.0 [60.0, 74.5]	0.001
Sex (male), *n* (%)	587	106,320 (64.7)	90,455 (64.7)	2855 (66.5)	0.019
Diabetes, *n* (%)	0	35,001 (21.2)	30,169 (21.6)	1002 (23.3)	0.007
Previous stroke, *n* (%)	0	4260 (2.6)	3595 (2.6)	135 (3.1)	0.024
Previous MI, *n* (%)	0	42,593 (25.8)	37,119 (26.5)	811 (18.8)	<0.001
Previous PCI, *n* (%)	0	52,549 (31.9)	45,883 (32.8)	820 (19.1)	<0.001
Previous CABG, *n* (%)	0	8465 (5.1)	7301 (5.2)	191 (4.4)	0.025
Smoking status, *n* (%)	0	28,729 (17.4)	24,819 (17.7)	933 (21.7)	<0.001
Hypertension, *n* (%)	0	113,095 (68.6)	97,198 (69.5)	2707 (62.9)	<0.001
Kidney disease, *n* (%)	0	8570 (5.2)	7425 (5.3)	227 (5.3)	0.953
COPD, *n* (%)	0	4660 (2.8)	4064 (2.9)	132 (3.1)	0.563
Diagnosis STEMI, *n* (%)	0	18,011 (10.9)	14,330 (10.2)	1538 (35.7)	<0.001
Diagnosis NSTEMI, *n* (%)	0	21,886 (13.3)	18,106 (12.9)	943 (21.9)	<0.001
Diagnosis UA, *n* (%) *	0	37,721 (22.9)	31,186 (22.3)	979 (22.8)	0.489
Diagnosis unstable angina, *n* (%) *	0	3864 (2.3)	3279 (2.3)	73 (1.7)	0.006
STEMI direct transport, *n* (%)	21,596	4556 (3.2)	3594 (3.0)	392 (9.8)	<0.001
NSTEMI direct transport, *n* (%)	21,596	1259 (0.9)	1005 (0.8)	93 (2.3)	<0.001
Cardiac arrest at baseline, *n* (%)	0	253 (0.2)	190 (0.1)	21 (0.5)	<0.001
Death during the procedure, *n* (%)	0	324 (0.2)	250 (0.2)	37 (0.9)	<0.001
STEMI time from pain to first contact ≤ 2 h, *n* (%)	0	6919 (4.2)	5529 (4.0)	650 (15.1)	<0.001
STEMI time from pain to first contact > 2 h and ≤12 h, *n* (%)	0	13,505 (8.2)	10,815 (7.7)	1243 (28.9)	<0.001
STEMI time from pain to first contact > 12 h, *n* (%)	0	2087 (1.3)	1627 (1.2)	212 (4.9)	<0.001
STEMI time from first contact to inflation ≤ 2 h, *n* (%)	0	9746 (5.9)	7861 (5.6)	843 (19.6)	<0.001
STEMI time from first contact to inflation > 2 h and ≤12 h, *n* (%)	0	13,241 (8.0)	10,627 (7.6)	1204 (28.0)	<0.001
STEMI Time from first contact to inflation > 12 h, *n* (%)	0	388 (0.2)	304 (0.2)	48 (1.1)	<0.001
NSTEMI time from pain to first contact ≤ 2 h, *n* (%)	0	4175 (2.5)	3441 (2.5)	243 (5.6)	<0.001
NSTEMI time from pain to first contact > 2 h and ≤12 h, *n* (%)	0	14,514 (8.8)	12,097 (8.6)	749 (17.4)	<0.001
NSTEMI time from pain to first contact > 12 h, *n* (%)	0	14,196 (8.6)	11,836 (8.5)	720 (16.7)	<0.001
NSTEMI time from first contact to coronarography ≤ 2 h, *n* (%)	0	2767 (1.7)	2237 (1.6)	201 (4.7)	<0.001
NSTEMI time from first contact to coronarography > 2 h and ≤12 h, *n* (%)	0	14,561 (8.8)	12,147 (8.7)	753 (17.5)	<0.001
NSTEMI time from first contact to coronarography > 12 h, *n* (%)	0	5204 (3.2)	4434 (3.2)	191 (4.4)	<0.001
Number of vaccinations, mean (SD)	0	9529.3 (11,504.0)	8901.8 (10,584.9)	9402.6 (10,470.8)	0.002

CABG—coronary artery bypass graft; COPD—chronic obstructive pulmonary disease; MI—myocardial infarction; NSTEMI—non-ST-elevation myocardial infarction; PCI—percutaneous coronary intervention; Q—quartile; STEMI—ST-elevation myocardial infarction; UA—unstable angina; * variables counted separately by the computer algorithm (but they should be interpreted together).

## Data Availability

The datasets generated during and/or analyzed during the current study are available from the corresponding author upon reasonable request.

## Supplementary Materials

The following supporting information can be downloaded at: https://www.mdpi.com/article/10.3390/jcm11185394/s1.
