# Supplementary material for: The Effect of COVID-19 on the Perioperative Course of Acute Coronary Syndrome in Poland: The Estimation of Perioperative Prognosis and Neural Network Analysis in 243,515 Cases from 2020 to 2021
Source: J Clin Med. 2022 Sep 14;11(18):5394. doi: 10.3390/jcm11185394 (PMC9506468; doi:10.3390/jcm11185394)
Supplement: Supplementary file 1 [file jcm-11-05394-s001.zip › jcm-1792504-supplementary.pdf]

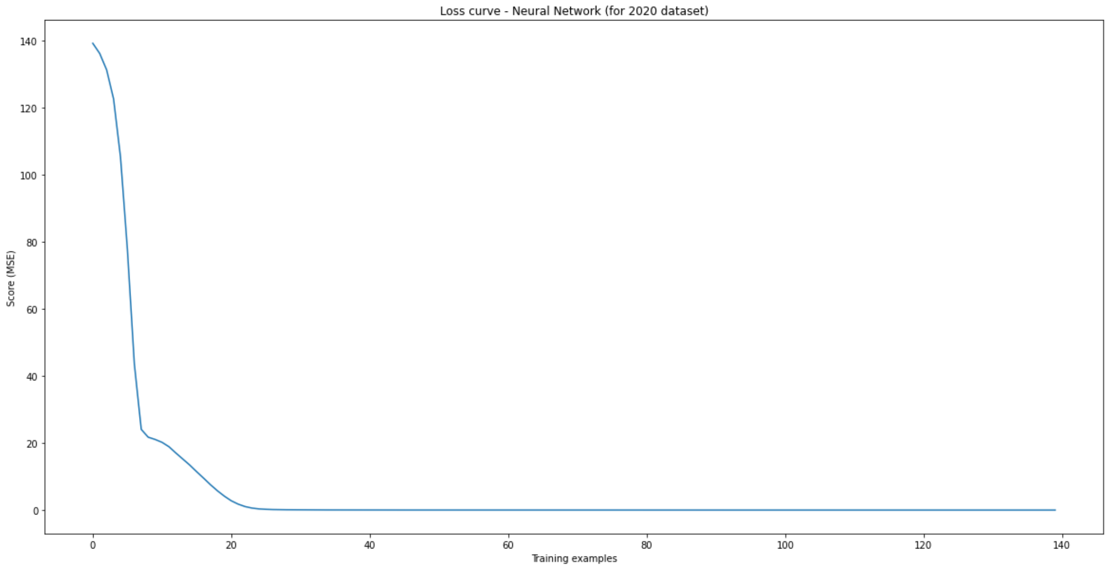

Supplementary Figure S1. Loss curve – Neural Network for patients in 2020.

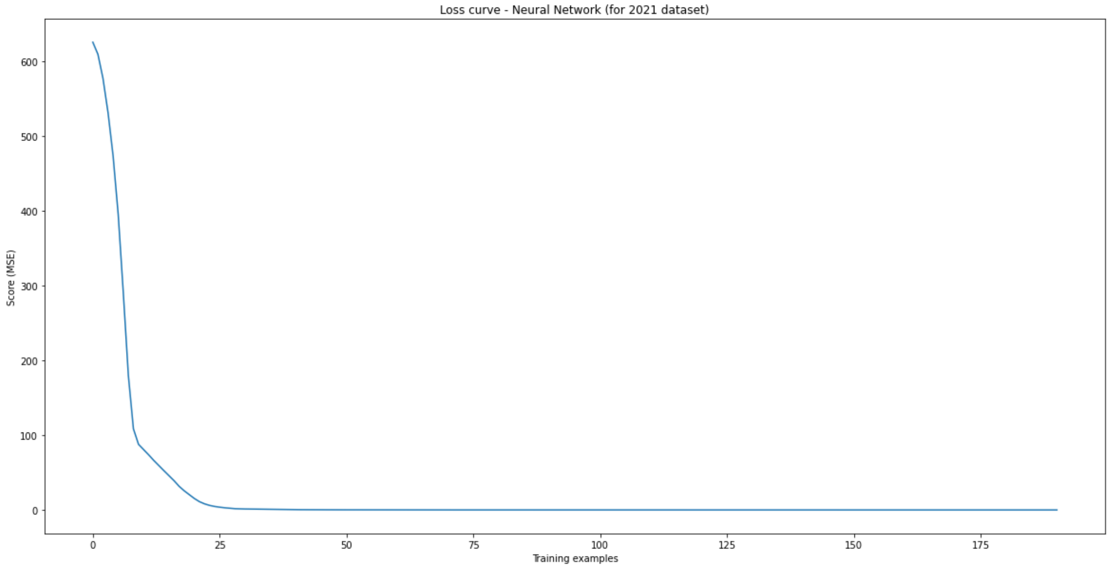

Supplementary Figure S2. Loss curve – Neural Network for patients in 2021.

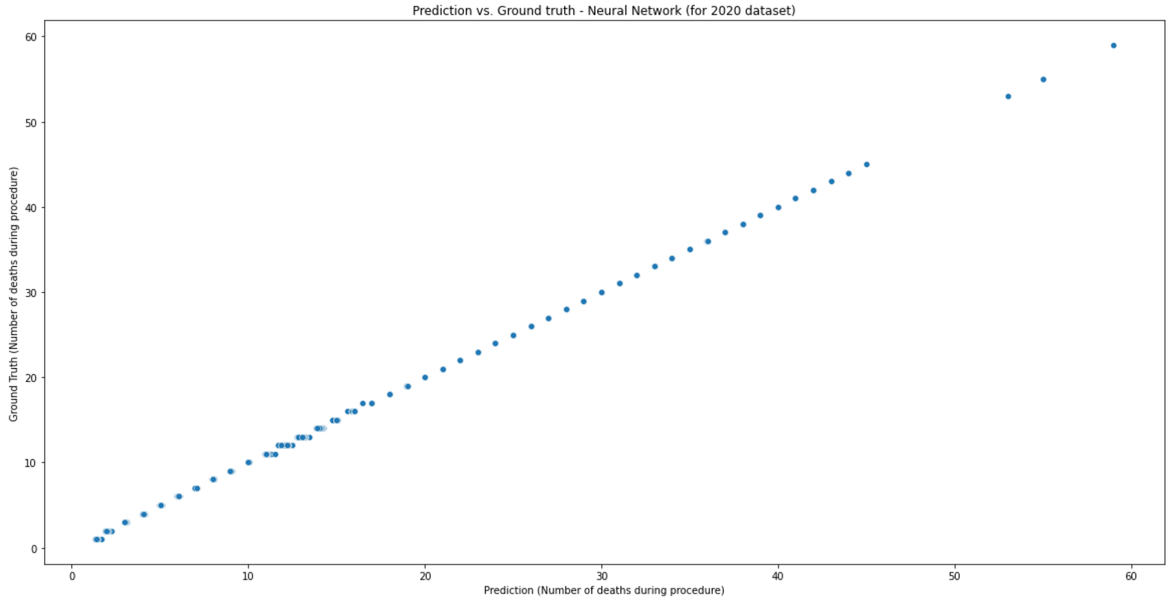

Supplementary Figure S3. Prediction vs. Ground truth – Neural Network for patients in 2020.

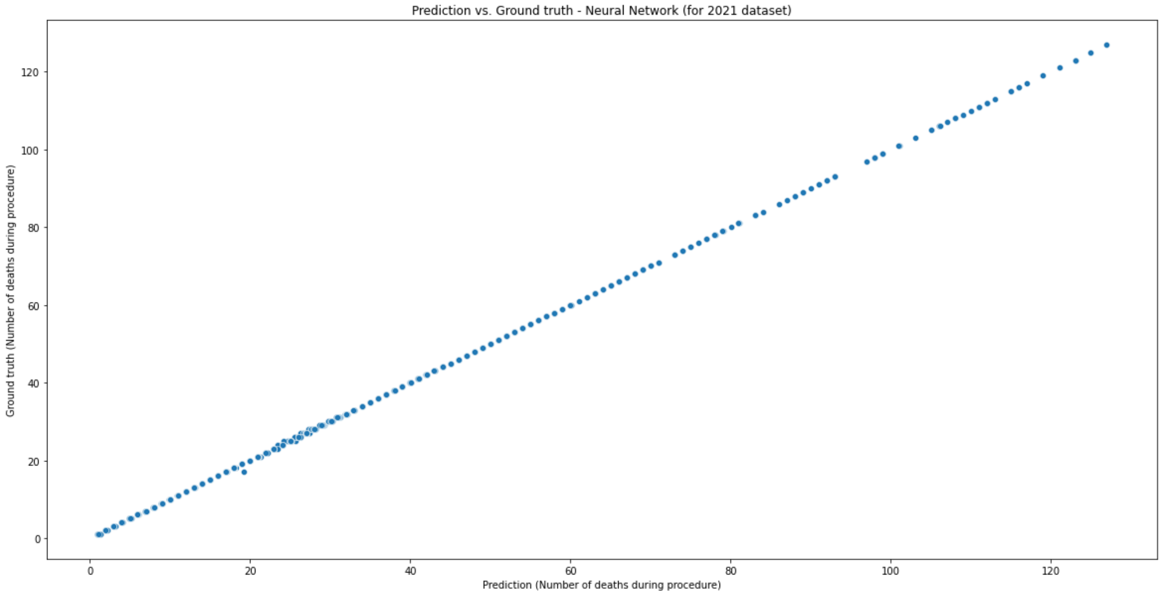

Supplementary Figure S4. Prediction vs. Ground truth – Neural Network for patients in 2021.

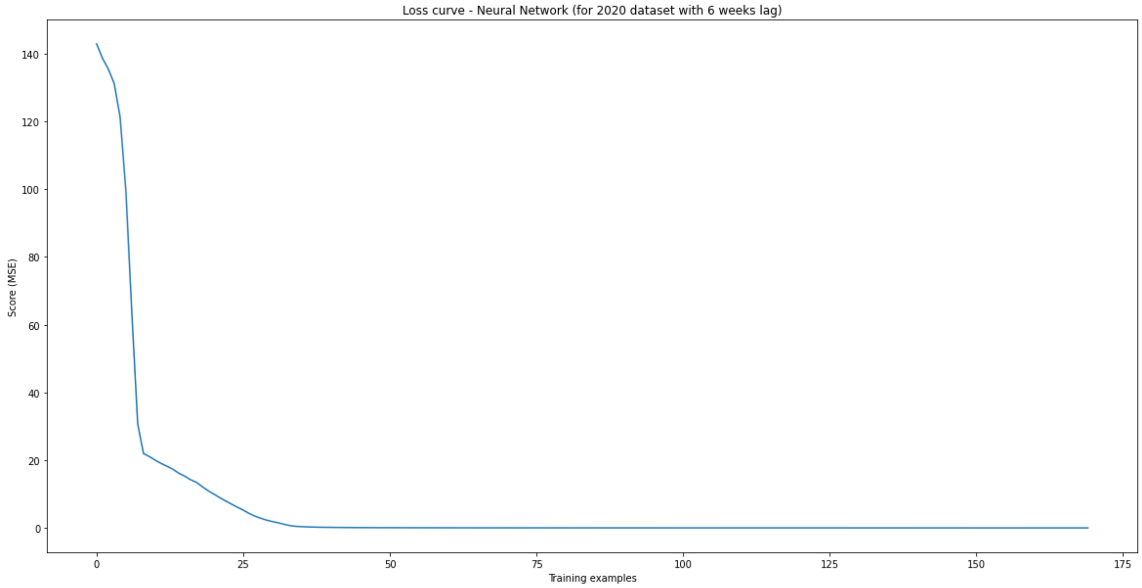

Supplementary Figure S5. Loss curve – Neural Network for patients in 2020 with six weeks lag.

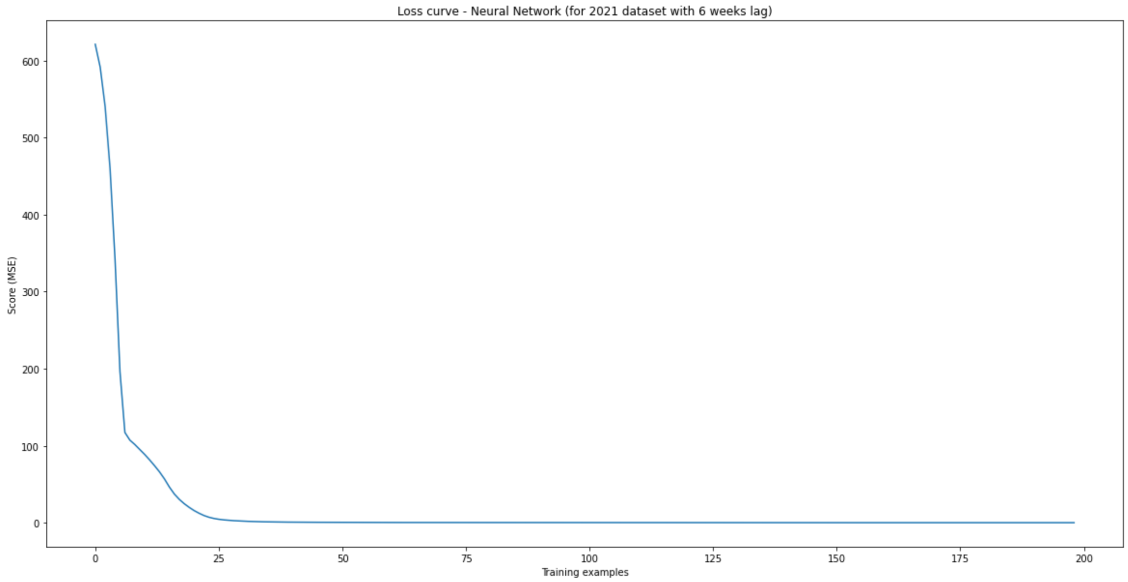

Supplementary Figure S6. Loss curve – Neural Network for patients in 2020 with six weeks lag.

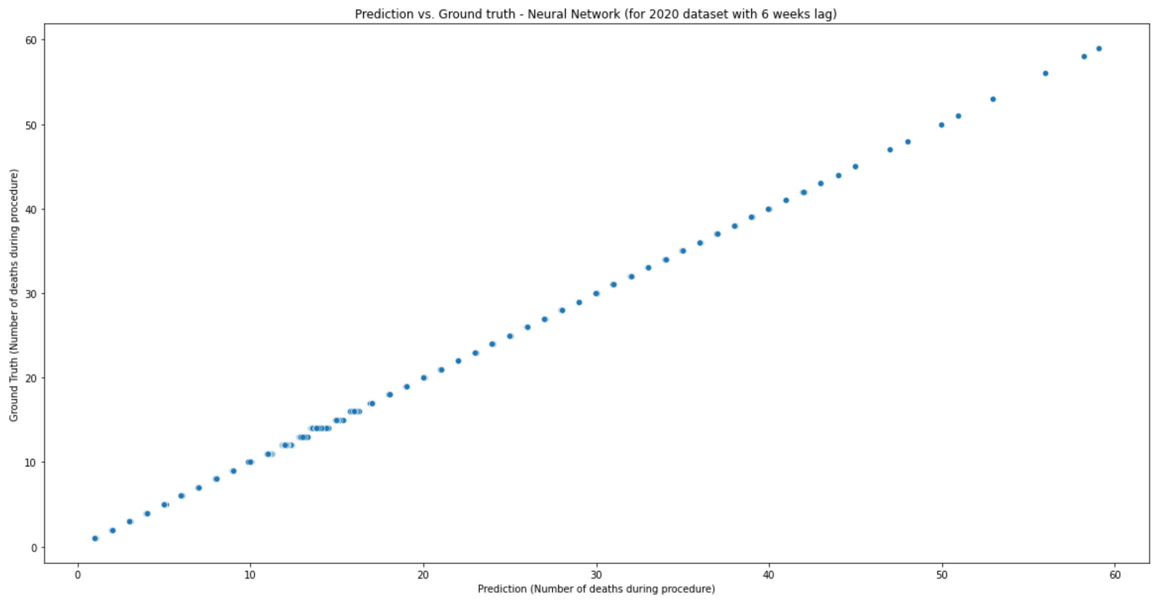

Supplementary Figure S7. Prediction vs. Ground truth – Neural Network for patients in 2020 with six weeks lag.

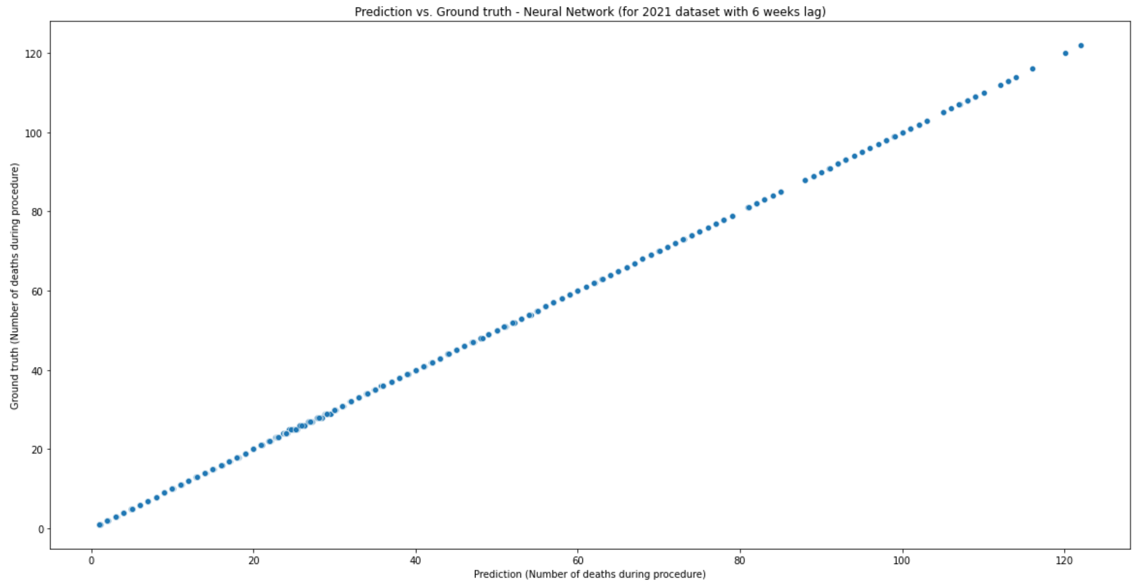

Supplementary Figure S8. Prediction vs. Ground truth – Neural Network for patients in 2021 with six weeks lag.

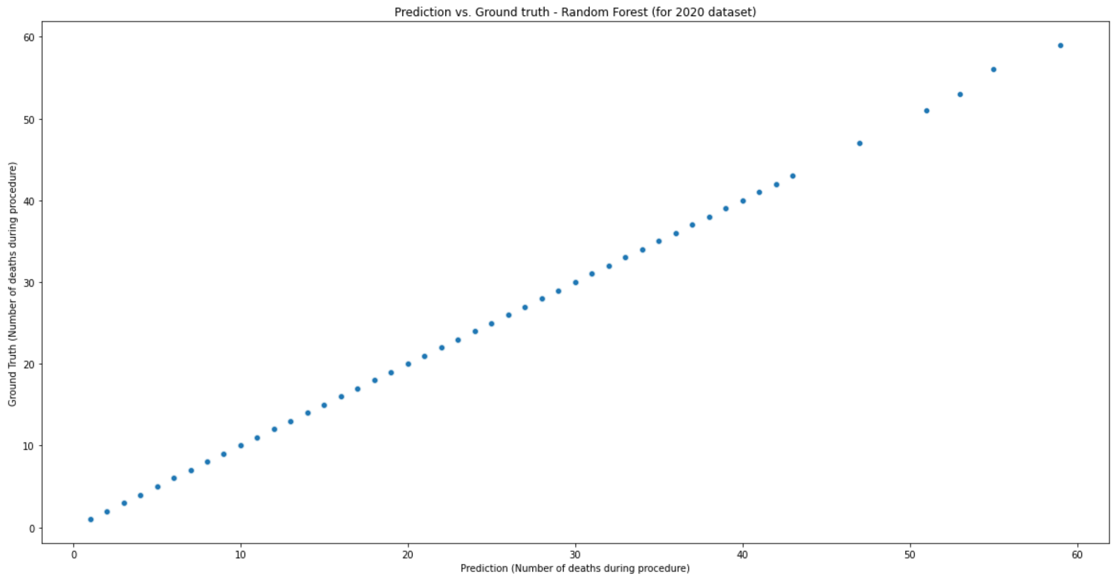

Supplementary Figure S9. Prediction vs. Ground truth – Random Forest for patients in 2020.

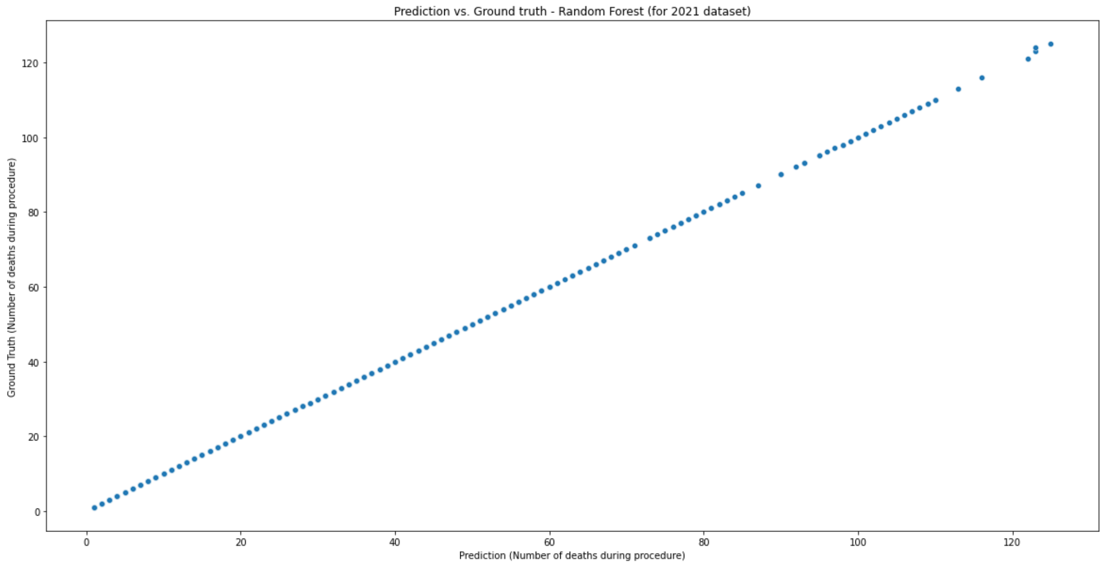

Supplementary Figure S10. Prediction vs. Ground truth – Random Forest for patients in 2021.

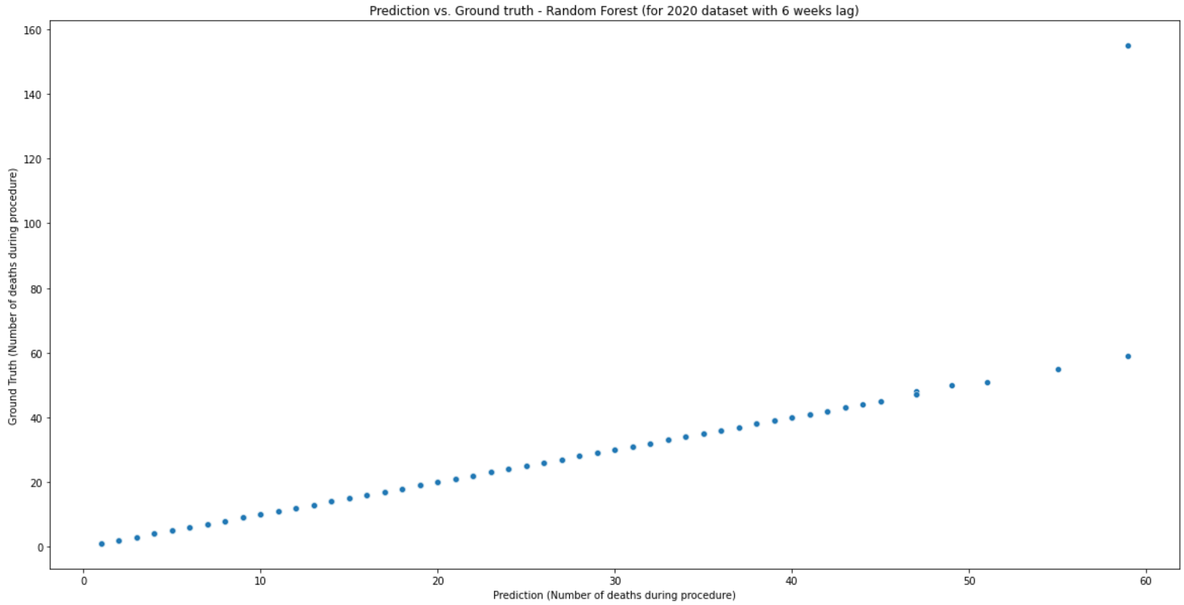

Supplementary Figure S11. Prediction vs. Ground truth – Random Forest for patients in 2020 with six weeks lag.

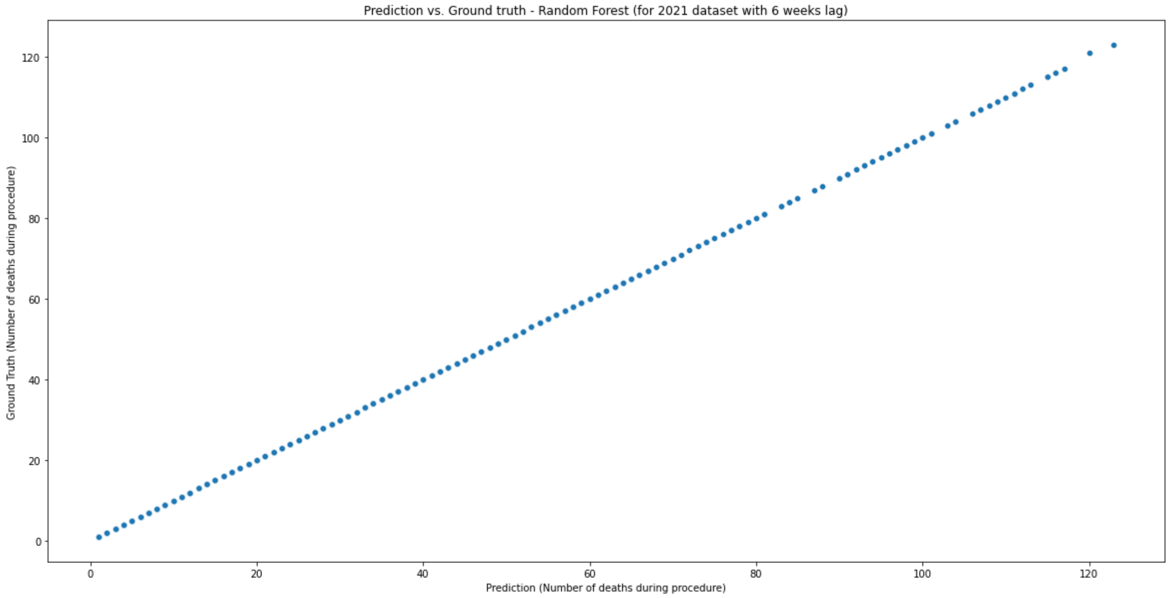

Supplementary Figure S12. Prediction vs. Ground truth – Random Forest for patients in 2021 with six weeks lag.
